# Use of a Decision Support Tool on Prognosis of Work Ability in Work Disability Assessments: An Experimental Study Among Insurance Physicians

**DOI:** 10.1007/s10926-020-09907-w

**Published:** 2020-06-11

**Authors:** I. Louwerse, M. A. Huysmans, H. J. van Rijssen, C. L. I. Gielen, A. J. van der Beek, J. R. Anema

**Affiliations:** 1 Department of Public and Occupational Health, Amsterdam Public Health Research Institute, Amsterdam UMC, Vrije Universiteit Amsterdam, Van der Boechorststraat 7, NL-1081 BT Amsterdam, The Netherlands; 2 Dutch Institute of Employee Benefit Schemes (UWV), Amsterdam, The Netherlands; 3 Research Center for Insurance Medicine, AMC-UMCG-VUmc-UWV, Amsterdam, The Netherlands

**Keywords:** Occupational health professionals, Evidence-based decision support tool, Prognosis of work ability, Prognostic confidence, Barriers and facilitators

## Abstract

*Purpose* Assessment of prognosis of work disability is a challenging task for occupational health professionals. An evidence-based decision support tool, based on a prediction model, could aid professionals in the decision-making process. This study aimed to evaluate the efficacy of such a tool on Dutch insurance physicians’ (IPs) prognosis of work ability and their prognostic confidence, and assess IPs’ attitudes towards use of the tool. *Methods* We conducted an experimental study including six case vignettes among 29 IPs. For each vignette, IPs first specified their own prognosis of future work ability and prognostic confidence. Next, IPs were informed about the outcome of the prediction model and asked whether this changed their initial prognosis and prognostic confidence. Finally, respondents reported their attitude towards use of the tool in real practice. *Results* The concordance between IPs’ prognosis and the outcome of the prediction model was low: IPs’ prognosis was more positive in 72 (41%) and more negative in 20 (11%) cases. Using the decision support tool, IPs changed their prognosis in only 13% of the cases. IPs prognostic confidence decreased when prognosis was discordant, and remained unchanged when it was concordant. Concerning attitudes towards use, the wish to know more about the tool was considered as the main barrier. *Conclusion* The efficacy of the tool on IPs’ prognosis of work ability and their prognostic confidence was low. Although the perceived barriers were overall limited, only a minority of the IPs indicated that they would be willing to use the tool in practice.

## Introduction

Individuals who are unable to work due to a disease or disorder can apply for a work disability benefit. In most European countries, this covers both financial support to compensate loss of income, and interventions to support return to work [1]. Occupational health professionals conduct work disability assessments to evaluate whether a benefit should be granted. One of the main tasks during this assessment is estimating prognosis of work ability [2]. Accurate prognosis is important to determine when an individual’s work ability will improve or deteriorate to such an extent that adjustment of the benefit or support to return to work is required [3]. However, it is also considered the most difficult part of the work disability assessment, because it requires rather complex predictions, in which a broad range of individual characteristics and external factors play a role [4, 5].

A potential solution to this problem is to provide occupational health professionals with evidence‐based decision support tools. These tools are comprised of software and designed to aid decision-making [6]. They match characteristics of individual claimants with a computerized knowledge base to generate patient-specific assessments or recommendations [7]. Although previous research has shown that such tools could be a means to achieve more accurate estimates of prognosis, they are usually based on a limited number of prognostic factors and are not 100% correct [8, 9]. In addition, other factors play a role in decision making in the medicolegal setting of work disability assessments. Hence, decision support tools are not meant to take over the job of professionals, but to support them by providing objective estimates of outcome probabilities to complement their professional expertise, competencies and experience. For instance, a decision support tool based on an evidence-based prediction model for future changes in work ability could aid professionals during work disability assessments [10, 11]. The tool could help professionals to make more precise estimations of future work ability and could increase their prognostic confidence [12]. To establish the possible efficacy of the decision support tool on the complex decision-making processes during work disability assessments, insight in actual use of the tool and occupational health professionals’ attitudes towards such a tool is needed.

In general occupational health professionals recognize the potential usefulness of evidence-based decision support tools. However, previous studies have shown that adherence to the use of innovations in medical settings can be a difficult task to accomplish [13]. Many factors may influence the use of decision support tools in practice, such as lack of knowledge about the innovation, negative attitudes and beliefs towards the innovation, perceived lack of time, lack of motivation, and organizational constraints [14, 15]. Moreover, barriers operate on different levels: they can be related to the professional, the patient, the organization, and the social and cultural context [16]. Insight into the barriers and facilitators for use of the decision support tool is needed to be able to further develop the tool in line with professionals’ needs and select an appropriate strategy for future implementation [17].

The objectives of our study were: (i) to evaluate the efficacy of the decision support tool on Dutch insurance physicians’ (IPs) prognosis of work ability using case vignettes; (ii) to investigate whether use of the tool affects IPs’ prognostic confidence; and (iii) to quantitatively assess the attitudes of professionals towards a decision support tool, and the perceived barriers and facilitators for use.

## Methods

An experimental study including six case vignettes was conducted to answer the research questions.

### Context

In the Netherlands, IPs conduct medical disability assessments to evaluate whether a work disability benefit should be granted. Once a work disability benefit has been granted, changes in work ability may alter its continuing eligibility. Therefore, prognosis of future changes in work ability is an important task of IPs [18]. IPs conduct medical re-assessments to determine whether a claimant’s health has improved or deteriorated to such extent that adjusting the benefit and/or support to return to work is necessary. Re-assessments are not only an operational aspect of the disability system, but a means to monitor claimants’ functional abilities. Depending on the situation of individual claimants, the term or the extent of the financial support of the benefit could be changed, or new rehabilitation interventions could be offered. Claimants could have interest in a certain outcome of a re-assessment. However, IPs are trained in objective assessment of functional limitations; IPs do not mainly focus their assessment on claimants’ self-perceived health complaints and impairments but they use many other factors as well [19–21].

During the work disability assessment, IPs need to indicate for each claimant if and when a re-assessment should be planned. Because of the large number of work disability claimants and the limited capacity to perform re-assessments, accurate prognosis is important for efficient planning of medical re-assessments and adequate interventions to support return to work. An evidence-based prediction model could help IPs in making more accurate prognosis of individual claimants during the work disability assessment. The prediction model is a regression equation that uses some prognostic factors to predict for each claimant the expected change in work ability at one-year follow-up [10]. A cohort of 944 claimants who were granted a work disability benefit by the SSI was used to develop the prediction model and for internal validation of the model [21]. Work ability was measured using the Work Ability Score (WAS), a single item of the Work Ability Index (WAI) questionnaire that asks participants to compare their current work ability with their lifetime best on a 0–10 scale [22]. Higher scores indicate better work ability, and an improvement or a deterioration in WAS of at least two points is considered to be a relevant change likely to have an effect on return to work and work disability benefit [23, 24]. Based on the predicted change in WAS at one-year follow-up, claimants are divided into three groups: claimants with no relevant change, an improvement, or a deterioration in the expected level of work ability at one-year follow-up compared to baseline. The prognostic factors of the prediction model are several physical and mental functioning factors, work status, wage loss, and work ability at baseline.

In order to make the outcome of the prediction model easily accessible and interpretable for professionals it needs to be supported by a suitable interface, i.e. a decision support tool. Based on professionals’ preferences regarding the way of use and design of the tool, we developed such a tool [11]. The tool uses claimant-specific information from self-reported questionnaires and registration data from the Dutch Social Security Institute (SSI) [21]. This information is matched with a decision rule, and presents the predicted change in work ability. IPs could use the tool as an auxiliary source of information to estimate prognosis of work ability, guide return to work interventions and plan re-assessments.

### Study Design

To assess the efficacy of using the decision support tool and IPs attitudes towards the tool, we conducted an experimental study including six case vignettes based on past work disability assessment reports [25, 26]. Advantages of vignette studies are that they are more realistic and less abstract than traditional survey questions, and that they can give insight into decision-making processes in an experimental setting [27, 28]. In consultation with three IPs working at the research department of the SSI and based on real patients’ records, we constructed detailed descriptions of six claimants who were granted a work disability benefit. These descriptions included demographic factors, information about the last job (working hours, work demands), disorders, medical and non-medical treatments, and functional limitations. All variables that were included as prognostic factors in the prediction model were also presented to IPs in the case vignettes. Table 1 shows a summary of the six case vignettes. The case vignettes were presented to respondents for evaluation. Cases were selected in such a way that several factors believed to influence the judgement were varied, e.g. demographics, disease type, disease history, and predicted change in work ability at one-year-follow-up. As an example, we have added a more detailed description of one of the case vignettes to the Appendix. The six cases were considered to sufficiently represent the most important factors in work disability assessments and the possible outcomes of the prediction model. As we did not want to pose an unnecessary burden on the already limited resources at the SSI, we decided not to include any additional cases.

**Table 1:** Main characteristics of the six case vignettes

Vignette	Gender	Age	Job demands	Disorder(s)	Type of limitations	Functioning and treatment	Self-assessed WAS^a^ at baseline
1	Male	45	Mostly physical	Paraplegia	Physical	ADL dependence	1
2	Female	29	Mental + physical	Chronic kidney disease	Physical	Waiting list for a kidney transplant	1
3	Male	34	Mostly physical	First episode psychosis	Mental	Hospitalized for treatment	4
4	Male	58	Mental + physical	Cerebral bleed	Mental + physical	Psychologist, occupational therapist	3
5	Female	38	Mostly mental	Depression, PTSD	Mental	Psychotherapy and EMDR	0
6	Female	34	Mostly mental	PTSD, multiple fractures	Mental + physical	Psychologist, physiotherapist	0

^a^*WAS* work ability score

### Study Population

The study population consisted of IPs working at the SSI. The SSI is a semi-governmental organisation that assesses sickness absence and work disability benefit claims, takes care of benefit payments and provides reintegration support. Seven of the 27 offices of the SSI were selected based on their willingness to participate and geographical distribution: three in the North, two in the central part and two in the South of the Netherlands. At each office, a meeting was organised to inform IPs about the design and questionnaires of the experimental study, and to give them more information about the prediction model and decision support tool. Thereafter, IPs were asked if they were willing to participate in the study and were invited per e-mail to fill in an online questionnaire. Participation was voluntary. Inclusion criteria were being registered as an IP or following the postgraduate education in insurance medicine, and conducting medical disability assessment interviews of work disability claimants. All participants signed informed consent and all data were anonymized. The Medical Ethics Committee of VU University Medical Centre in Amsterdam, The Netherlands, has confirmed that ethics approval is not necessary, because the Medical Research Involving Human Subjects Act (WMO) does not apply to our study.

### Questionnaire

The questionnaire consisted of three sections: a general section, evaluation of the six case vignettes, and a section to evaluate aspects for use of the tool. The general section included questions about demographics and professional characteristics such as age, gender, and work experience. Next, the six case vignettes were presented. For each vignette, IPs were asked about their prognosis of future changes in work ability based on a detailed description of demographic, work, health and psychological factors. Based on this prognosis, they were asked to specify duration of the period after which they wanted to plan a re-assessment and the expected change in work ability at one-year follow-up. In line with the outcome of the prediction model, the latter question had three answering categories: improvement, no change, or deterioration of a claimant’s work ability. Moreover, IPs were asked to rate the level of prognostic confidence on a numerical rating scale (NRS), ranging from 0 (no confidence) to 10 (complete confidence). Next, the decision support tool was presented showing the prognostic factors and the outcome of the prediction model. The prognostic factors were also included in the detailed description of the case vignettes, but were presented again to enable IPs to judge the exact factors that were used to estimate the predicted outcome. IPs were asked to re-evaluate their prognosis using the same description of the case vignettes and the outcome of the prediction model. IPs indicated whether consulting the tool led them to change their prognosis or not, and if so in which direction (i.e. better or worse). An open-ended question gave respondents the opportunity to explain why they did or did not change their prognosis. Besides, to assess whether the tool made them more or less confident about their prognosis, respondents again indicated their prognostic confidence on a numerical rating scale.

To study future use of the decision support tool, IPs were asked if and how often they considered the tool to be of benefit during the medical disability assessment. Moreover, it was examined whether IPs would consider using a decision support tool in the future to support their prognosis during the work disability assessment, how they would want to use the tool, and in what situations or for which types of claimants they would not use it. Finally, 15 statements about barriers and facilitators for use of a decision support tool in real practice were presented. These statements originate from an existing validated questionnaire and assess constructs related to the society, the organization, the claimant, the future user of the decision support tool, and the tool itself [29]. A 5-point Likert scale ranging from “Fully disagree” to “Fully agree” was used to rate the extent of agreement with each statement.

### Statistical Analysis

Descriptive statistics were used to describe the demographic and professional characteristics of the IPs (means, standard deviations, percentages). We assessed the efficacy of the decision support tool on the IPs’ prediction of prognosis. Prognosis was defined as either concordant or discordant according to whether the prediction of the decision support tool was or was not equal to IPs’ own prediction of prognosis, i.e. before evaluating the decision support tool. To measure whether use of the decision support tool led IPs to change their prognosis, the number and percentages of IPs in the three answering options (i.e. prognosis of work ability remained unchanged, got worse, or got better after evaluating the decision support tool) were calculated. Differences between cases with different types of limitations (mental, physical, or both mental and physical) were assessed using chi-square tests. For each case vignette, we calculated the proportions of observed agreement between IPs’ prognosis and the outcome of the prediction model and used McNemar's test for paired proportions to compare agreement before and after evaluating the decision support tool. Multilevel analyses were used to assess changes in the level of prognostic confidence after consulting the decision support tool, taking into account that the data were clustered within IPs. Concerning the statements on barriers and facilitators for use, we calculated the percentage “agree” and “fully agree” for all statements to identify possible barriers and facilitators for use of the decision support tool. All analyses were performed in RStudio for Windows, version 0.99.902. The significance level of all statistical tests was set at *p* < 0.05.

## Results

Twenty-nine IPs voluntarily participated in the study. This was just above the minimum number of 28 IPs that was determined in a sample size calculation as number needed to answer the research questions. Table 2 summarizes the demographic and professional characteristics of the respondents. The majority was female (59%) and worked as a registered IP (62%).

**Table 2:** Demographic and professional characteristics of the respondents (n = 29)

	n	%	Mean (SD)
Gender			
Male	12	41	
Female	17	59	
Age (years)			44 (11)
< 35	7	24	
35–44	10	34	
45–54	4	14	
55 +	8	28	
Type of IP			
Registered	18	62	
Postgraduate student	11	38	
Working experience as IP (years)			12 (11)
< 5	10	34	
5–9	7	24	
10 +	12	41	

### Prognosis Without and With the Decision Support Tool

In 82 (47%) cases, the prognosis of the IP without information from the decision support tool was concordant with the outcome of the prediction model (Table 3). The prognosis of the IP was more positive in 41% (*n* = 72) and more negative in 11% (*n* = 20) of the cases. Differences in prognosis occurred most often in cases with both mental and physical limitations (90%) and least often in cases with only physical limitations (26%).

**Table 3:** Prognosis without and with the decision support tool (DST) in 29 IPs judging 6 case vignettes

	Vignette						
	1 (n = 29)	2 (n = 29)	3 (n = 29)	4 (n = 29)	5 (n = 29)	6 (n = 29)	All (n = 174)
Change in WAS predicted by DST^a^	No change	Improve	No change	Deteriorate	Improve	No change	-
Without the DST^a^							
Prognosis, *n* (%)							
Improvement	0 (0)	14 (48)	20 (69)	0 (0)	24 (83)	24 (83)	82 (47)
No change	29 (100)	10 (35)	9 (31)	28 (97)	5 (17)	5 (17)	86 (49)
Deterioration	0 (0)	5 (17)	0 (0)	1 (3)	0 (0)	0 (0)	6 (4)
Concordant prognosis, *n* (%)	29 (100)	14 (48)	9 (31)	1 (3)	24 (83)	5 (17)	82 (47)
Observed agreement, %	100	48	31	3	83	17	47
With the DST^a^							
Prognosis, *n* (%)							
Improvement	0 (0)	17 (59)	14 (48)	0 (0)	25 (86)	17 (59)	73 (42)
No change	29 (100)	9 (31)	15 (52)	26 (90)	4 (14)	12 (41)	95 (55)
Deterioration	0 (0)	3 (10)	0 (0)	3 (10)	0 (0)	0 (0)	6 (3)
Changed prognosis, *n* (%)	0 (0)	3 (10)	6 (21)	2 (7)	1 (3)	7 (24)	19 (11)
Observed agreement, %	100	59	52	10	86	41	58
Change in IPs’ prognosis^b^, *p*-value	NS	NS	0.026	NS	NS	0.008	-

^a^*DST* decision support tool

^b^McNemar’s test to compare IPs’ prognosis before and after use of the decision support tool, *NS* not significant (*p* > 0.05)

For all cases where the prognosis of the IP was concordant with the outcome of the prediction model, IPs did not change their prognosis after evaluating information from the decision support tool. In 22% (*n* = 20) of the cases where the prognosis was not concordant, the IP changed his or her prognosis after evaluating information from the tool. In the majority of the cases where the prognosis was changed, IPs considered the prognosis to be worse after evaluating the decision support tool (75%; *n* = 15). Whether or not the IP changed his or her prognosis was independent of the type of limitations (*p* = 0.34).

Overall, the observed agreement between IPs’ prognosis and the outcome of the prediction model increased from 47 to 58% after IPs evaluated the decision support tool. If we look at each of the case vignettes separately, we can see that for two of the vignettes that showed low initial agreement, there was an improvement in agreement after IPs were informed about the outcome of the prediction model. For these vignettes, the prediction model estimated no change in future work ability. There was a statistically significant change in the number of IPs that first predicted an improvement or deterioration in work ability and, after use of the tool, agreed with the outcome of the prediction model.

### Confidence in the Prognosis With and Without the Decision Support Tool

Table 4 presents the prognostic confidence of IPs without and with information from the decision support tool. In 57% (*n* = 99) of the cases, the prognostic confidence of the IP changed after evaluation of the decision support tool. The confidence increased in 26% (*n* = 45) and decreased in 31% (*n* = 54) of the cases. Change in prognostic confidence occurred less often when the prognosis of the IP was concordant with the outcome of the decision support tool (*n* = 40; 49%) than in case of discordant prognosis (*n* = 59; 64%).

**Table 4:** Efficacy of a decision support tool (DST) on prognosis of work ability and prognostic confidence

	All (n = 174)	Concordant prognosis (n = 82)	Discordant prognosis (n = 92)
Changed prognosis, *n* (%)	20 (13)	–	20 (22)
Prognostic confidence			
Without the DST^a^	7.1 ± 1.7	7.2 ± 1.5	7.0 ± 1.9
With the DST^a^	6.6 ± 2.2	7.4 ± 1.6	6.0 ± 2.4
Δ NRS^b^	− 0.5 ± 2.1*	0.2 ± 1.1	− 1.0 ± 2.5*

^a^*DST* decision support tool, ^b^*NRS* numerical rating scale

^*^ Significant decrease in prognostic confidence after using the DST (*p* < 0.05)

The results of the multilevel analyses showed that there was an overall decrease in prognostic confidence by 0.5 points on the NRS from 7.1 to 6.6 points (*p* = 0.02). If the prognosis was concordant, then the prognostic confidence increased from 7.2 to 7.4 points, but this increase was not statistically significant (*p* = 0.26). The prognostic confidence significantly decreased from 7.0 to 6.0 points in cases with discordant prognosis (*p* < 0.001).

### Use of the Decision Support Tool in Practice

28% (*n* = 8) of the respondents indicated that they would be willing to use an evidence-based decision support tool based on a prediction model during future work disability assessments. The majority of the respondents was unsure (55%; *n* = 16), and 17% (*n* = 5) indicated that they would probably not be willing to use the tool. The responding IPs were more negative about the attitudes of their colleagues: 90% (*n* = 26) doubted whether their colleagues would be willing to use a decision support tool during work disability assessments. Respondents expected that the tool would be of most benefit for claimants with more complex pathology, such as medically unexplained physical symptoms, in which motivation and perception play an important role. Regarding the question for which types of claimants the tool would be of less added value, respondents mentioned claimants of which the prognosis is evident (55%) and claimants who lack insight into their own illness (21%). However, 28% of the respondents did not specify specific types of claimants and indicated that the decision support tool could always be consulted. These were not (all) the same respondents as the eight IPs that indicated who they would be willing to use an evidence-based decision support tool based on a prediction model during future work disability assessments.

### Barriers and Facilitators for Use of the Decision Support Tool

**Fig. 1:**
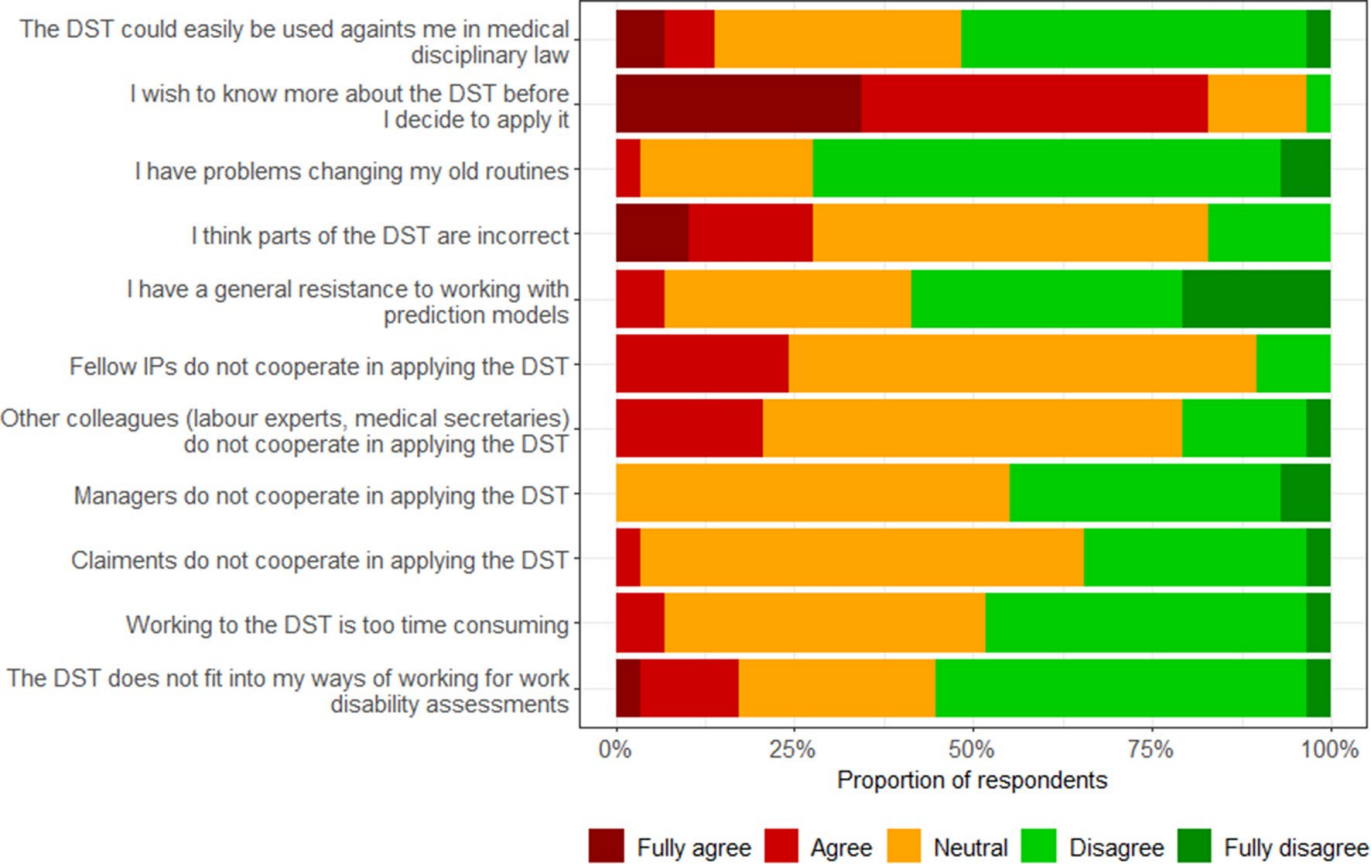
Barriers for use of the decision support tool (DST)

**Fig. 2:**
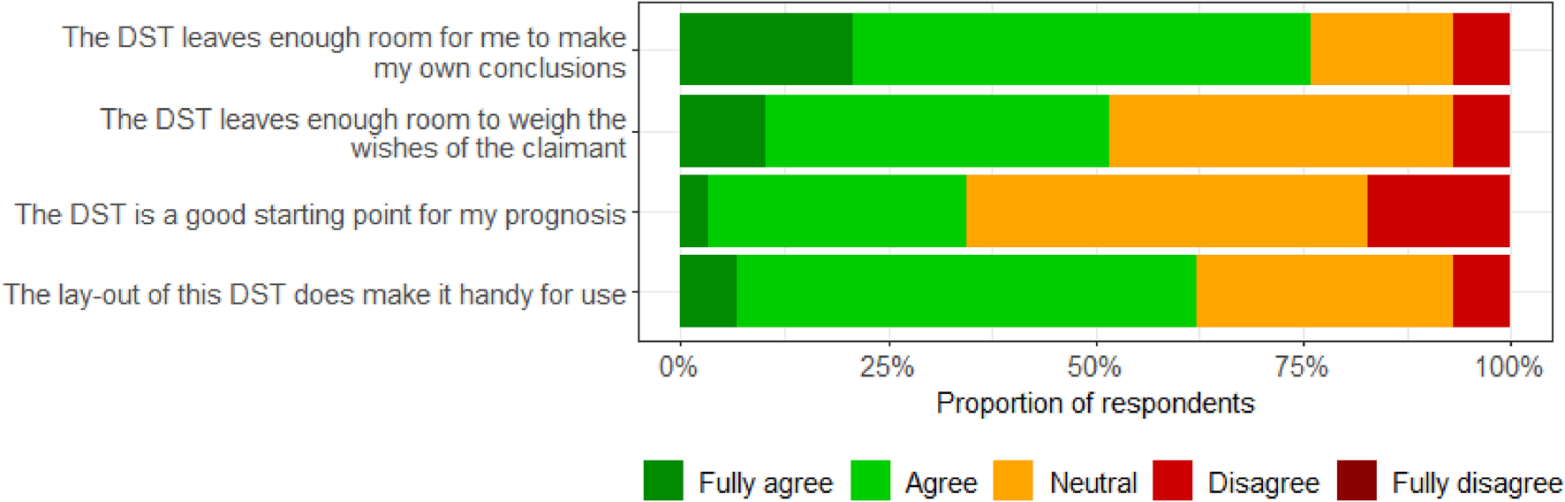
Facilitators for use of the decision support tool (DST)

The percentages of respondents that agreed, neither agreed nor disagreed, and disagreed that specific barriers or facilitators applied to using of the decision support tool in practice are summarized in Figs. 1 and 2, respectively. Among the barriers, wishing to know more about the decision support tool before deciding to apply it showed the highest score (83%). Other barriers were thinking that parts of the decision support tool are incorrect (28%), and that fellow IPs (24%) and other colleagues (21%) would not cooperate in applying the tool. Overall, the mean percentages of IPs who agreed that certain facilitators were applicable to use of the tool were somewhat higher. Concerning the facilitators that were applicable to use of the decision support tool, the majority of the respondents agreed that the tool leaves them enough room to make their own decision (76%), that the layout of the tool makes it handy for use (62%), and that it leaves enough room to weigh the wishes of the claimant (52%).

## Discussion

We found that in only 47% of the cases the prognosis of the IP without information from the decision support tool was concordant with the outcome of the prediction model. In 22% of the cases with a discordant prognosis, IPs changed their prognosis of work ability after consulting the decision support tool. If IP’s prognosis was discordant with the outcome of the decision support tool, their prognostic confidence decreased. Although the perceived barriers for use were overall limited, only a minority of the IPs (28%) indicated that they would be willing to use an evidence-based decision support tool based on a prediction model in practice.

In the present study, we found that the agreement between IPs’ own prognosis before evaluating the outcome of the decision support tool and the outcome of the prediction model was low. In general, prognosis of future changes in work ability was more positive reported by the IPs than the prognosis estimated by the prediction model. As IPs consider prognosis as the most difficult part of the work disability assessment, this could indicate that IPs often give claimants the benefit of the doubt and, when they are unsure, prefer to plan re-assessments to monitor claimants’ change in work ability.

In case IPs’ own prognosis was discordant with the outcome of the decision support tool, IPs changed their prognosis in 22% of the cases after they evaluated the tool. This was only 13% of the total number of cases. This low efficacy can possibly be explained by the low initial agreement between IPs’ prognosis and the outcome of the decision support tool, which could have influenced IPs’ trust in the tool. Although the percentage of changed prognoses in our study was rather low, this is in line with results from a study on the impact of a decision support tool on prediction of progression in early-stage dementia [30]. This prospective multicenter study including 429 patients showed that clinicians changed the prediction of progression only in 13% patients after using the tool. However, the researchers did not mention the number of predictions discordant with the outcome of the decision support tool, and this might have been lower than 53%. A study on Dutch IPs judgement of physical work ability showed that when IPs judgement differed from the information about functional tests, only a one-third changed their judgement [31].

IPs’ own prognosis was discordant with the outcome of the prediction model in 53% of the cases, but only in 13% of the cases, they changed their prognosis after consulting the tool. In a previous study using retrospective data, we have shown that the prediction model for future changes in work ability can discriminate between claimants with an improvement, deterioration or no change in work ability at one-year follow-up [10]. Internal validation of the prediction model showed that the positive and negative predictive values of the model were 63% and 82%, respectively. Compared to these percentages, the number of cases for which IPs’ prognosis was discordant with the outcome of the prediction model, but for which IPs did not change their prognosis (n = 73; 42%), was relatively high. This implies that for part of these cases for which the final prognosis of the IP was discordant, the prediction model might have correctly predicted the change in work ability at one-year follow-up. This could be an indication that there is still room for improvement in that IPs could have had more trust in the decision support tool and could have adjusted their prognosis accordingly. However, also in previous studies it was shown that clinicians seem to be reluctant to use decision support tools and other new sources of information in practice [32, 33]. Even if clinicians trust the scientific evidence underlying a decision support tool, they perceive it mostly as a confirmatory tool that can be used to validate or fine-tune their own prognosis rather than substantially change it [12].

High prognostic confidence is important for efficient planning of medical re-assessments and to provide effective interventions for return to work. Mean prognostic confidence without the decision support tool was 7.1 (SD 1.7). For concordant cases, our results did not show a significant change in prognostic confidence. For discordant cases, however, we found a statistically significant decrease of 1 point (SD 2.5). These confidence levels are in line with clinicians’ confidence in the prediction of dementia without and with a decision support tool, which ranged between 62–76 (SD 16–19) on a visual analogue scale (0–100%) [34]. In the latter study, they did not discriminate between concordant and discordant cases, and overall a small significant increase in confidence of 3% was found (SD 11). This is in contrast with the significant decrease in confidence of 1 point on the NRS found in the present study.

Transferring effective innovations into real world (medical) settings and achieving sustainable use in every day decision-making processes is a complicated, long‐term process [16, 35, 36]. We assessed 15 statements focusing on barriers and facilitators related to knowledge, attitude, and behavior. The majority of the respondents indicated that they currently doubted whether they would use the decision support tool for prognosis of work ability during the work disability assessment. Overall, the reported barriers among our IPs were limited. The main perceived barrier was that IPs felt that they needed more information about the decision support tool before they could decide whether to use the tool in practice. Lack of knowledge was also mentioned as one of the main barriers for adherence to guideline recommendations in a study among Dutch general practitioners [37]. The need for more information could have influenced the perceived usefulness of the decision support tool, which is a key characteristic for acceptance and use [38, 39]. In the present study, this need is possibly reinforced by the low agreement between the outcome of the prediction model and IPs’ prognosis of future work ability, and the fact that IPs first had to estimate their own prognosis while the decision support tool was only presented after their decision-making process.

The most important facilitators for use of the decision support tool were as follows: the majority of IPs believed that the decision support tool leaves them enough room to make their own conclusions (80%) and that the layout of the tool was perceived as handy for use (67%). The former may have arisen from the fact that in the present study IPs were first asked to assess their own prognosis, and subsequently they were informed about the outcome of the decision support tool. The latter seems a favorable factor for future use of the tool, as clear graphical representation supports the understanding of the decision support tool and leads to better informed decision-making [40, 41].

### Strengths and Limitations

The main strength of our study is that we focused on the efficacy of and attitudes towards actual use of an evidence-based decision support tool that was developed for and in accordance with Dutch IPs. Moreover, because of the within-subject design, respondents could act as their own control group. For each case vignette, respondents were first asked to give their prognosis based on the case description, after which the decision support tool with the outcome of the prediction model was presented. The former prognosis and prognostic confidence can be considered as control data, and compared with the outcome measures after the respondents consulted the tool.

Another strength of the study was that the participants were recruited out of all potential users of the decision support tool. There are small differences in working procedures between offices, as well as differences between claimants’ populations. As IPs from seven different offices participated in this study, we were able to gather different perspectives on the benefits of the tool, and on possible barriers and facilitators for use. The number of IPs that voluntary wanted to participate in the study (n = 29) was higher than the minimum number of IPs. However, a potential selection bias was introduced, as participation was voluntary. This may have led to an increased sampling of like‐minded IPs who are interested in prediction models and the use of evidence-based decision support tools in practice.

A second limitation is that, for practical and ethical reasons, we were unable to perform the randomized controlled trial (RCT) that we originally planned and could therefore not externally validate the model in a new dataset. Instead, we had to rely on evaluation of the decision support tool in an experimental setting. Using case vignettes, we could not assess the prognostic accuracy of either the decision support tool or the prognosis of the IPs. Although the case vignettes were based on real work disability assessment reports, they were fictitious descriptions of claimants and tested in an experimental setting. Hence, this did not allow us to make any statement about the level of work ability at one-year follow-up. Moreover, performing an experimental study as a substitute for a RCT meant that we could not consider any learning effects. In general, users need some time to get comfortable with a change. As the use of predictive analytics and decision support tools in insurance medicine is rather limited, it could be expected that their impact will gradually increase over time.

### Implications for Research and Practice

The use of evidence-based decision support tools in occupational health could improve decision-making processes and quality of work disability assessments. Such tools can guide IPs when they are unsure about the prognosis, and unveil prognostic factors that are important to draw attention to in the return to work process. In particular, a decision support tool has the potential of a supportive effect when prognosis of work ability is unclear. The tool was considered to be of less benefit for claimants of which the prognosis is evident. In the present study, IPs first made their own prognosis and subsequently evaluated the decision support tool. Although this is in line with the results of a previous study, in which professionals indicated that they would first want to make their own prognosis and afterwards verify or adjust their evaluation based on the outcome of decision support tool, this might have lowered the efficacy of the tool [11]. Presenting the tool at the beginning of the decision-making process might increase its efficacy by making the tool part of this process. Once external validation of the prediction model has confirmed its accuracy, it could be given the same value as other sources of information that IPs use, such as medical guidelines, information from treating physicians and the medical history of the claimant.

Effectiveness of implementation processes is strongly associated with innovations being carefully implemented and free from serious implementation problems. Insight in the perceived barriers and facilitators for use in work disability assessments can be used to design the future implementation process of the decision support tool. Only a minority of the IPs that participated in this study indicated that they would use a decision support tool in practice. Unfamiliarity with prediction models and decision support tool was mentioned as the main barrier that may prohibit IPs from using such tools. At the start of the present study, we organized short meetings to give IPs more information about the prediction model and decision support tool, and to inform them about the design and questionnaires of the experimental study. However, these short meetings might not have been sufficient. Participants might wish to have more detailed information on the prediction model, for instance on the weights of the prognostic factors that were included in the regression equation. To improve knowledge, it may be useful to conduct more extensive information sessions on the use of prediction models and decision support tool in insurance medicine or incorporate these topics in training programs. Moreover, to build trust with future users, the decisions support tool should be validated in occupational health practice. Therefore, future research should pay attention to the effect of using the decision support tool in everyday practice, focusing on IPs prognosis of work ability and prognostic confidence in work disability claimants with different types of diagnoses.

Although individual participants were rather reluctant towards use of a decision support tool in real practice, it may be beneficial from an organizational perspective. IPs’ prognosis during the initial work disability assessment of a claimant is used to schedule subsequent re-assessments. Our results indicate that IPs’ assessment of prognosis is relatively positive. Given the limited capacity of occupational health resources, and especially a shortage in the number of IPs at the SSI, the SSI might want to minimize the re-assessments for claimants for which no relevant change in work ability is expected. A prediction model for prognosis can help to use the limited available capacity as effective as possible, i.e. to plan re-assessments for claimants who will benefit most from contact with an IP. In this regard, elaborate knowledge transmission and embedding the tool in working policies seem important factors. Using decision support tools at an organizational level involves some ethical and medicolegal considerations for professionals and policy makers. Hence, it should be emphasized that decision support tools are not meant as stand-alone or management tools, but as a complement to professionals’ own estimates of prognosis.

The findings of the present study could be used when developing other prediction models and decision support tools for occupational health professionals. Our tool focuses on prognosis of work ability for claimants who were sick-listed for two years. However, the longer individuals are absent from work, the less likely they are to return [42–44]. A prediction model for workers who have just recently been sick-listed can help occupational health professionals to target individuals at risk of long-term sickness absence and identify effective early interventions [45, 46]. From a rehabilitation point of view, such a tool could have more impact. Our findings on the attitudes of IPs towards prediction models and decision support tools include general aspects that could be helpful when developing such a tool.

### Concluding Remarks

The present study showed that the congruence of the decision support tool with IPs’ prognosis of future work ability was low, and that IPs’ prognostic confidence decreased after evaluating the tool if their prognosis was discordant with the outcome of the prediction model. Only a minority of the IPs changed their prognosis when it was discordant with the outcome of the tool. Most IPs indicated that they were unsure or they were not willing to use an evidence-based decision support tool based on a prediction model during future work disability benefits. Unfamiliarity with prediction models and decision support tool was mentioned as the main barrier that may prohibit IPs from using such tools.

## Appendix

### Example of a Case Vignette Used in the Study

Description of the sixth case vignette that was given to IPs for consideration:

- Until two years ago, Yvonne (45 years old) worked as an administrative assistant for 40 h a week. Her job had a heavy workload, and required high levels of concentration and multitasking.
- She had to call in sick at work after she had a collision while riding her bike. This accident caused multiple fractures on both arms and knee complaints. Her physiatrist indicated that Yvonne has humerus fractures on her left and right arm, and that she got a surgery with metal screws and plates. She follows a rehabilitation trajectory, but due to her mental disorders, this runs longer than expected.
- A letter from her treating psychologist states that Yvonne suffers from a post-traumatic stress disorder and a mood disorder. When she was a child, she suffered from child traumatic stress. This caused instability in her mood and personality development. Yvonne has regular consults with her psychologist, but so far, she rejects a medical treatment.
- During the work disability assessment interview, Yvonne looks vulnerable. She says that she experiences many mental and physical limitations in daily functioning. She thinks she is not yet ready to return to work, but expects that her limitations will reduce in the future.
- Yvonne has limitations related to personal and social functioning: she has difficulty concentrating, often experiences sensory overload, avoids conflicts, and is very sensitive to other people’s feelings. Due to her physical complaints, she cannot optimally use her musculoskeletal system without pain and has difficulties with dynamic movements (lifting, twisting, bowing, walking at work etc.). On medical grounds, she gets a restriction in working hours for maximum 4 h a day, 20 h a week.
- Yvonne has psychological and orthopedic treatment. Three times a week she has an appointment with her physiotherapist. It is expected that her medical conditions and functional possibilities will substantially improve in the future.

Prognostic factors that were taken into account in the prediction model:

- If you would give 10 points to your work ability during the best period in your life, how many points (between 0–10) would you give to your level of work ability of the past 2 weeks? *0 points*
- During the past 4 weeks, due to your physical health, did you achieve less than you would in your work other daily activities? *Yes*
- How often during the past 4 weeks, did you feel so down that nothing could cheer you up? *Often*
- How often during the past 4 weeks, did you feel calm and peaceful? *Seldom*
- Since the first day of sickness absence, how often have you visited a treating practitioner? *More than 10 times*
- Type of comorbidity*: comorbidity of physical and mental disorders*
- Expected change in work ability at one-year follow-up as predicted by the prediction model: *no expected change in work ability*
